# Evaluating Clinical Factors Including HPV Clearance on Survival Outcomes in HPV+ Oropharyngeal Carcinoma †

**DOI:** 10.3390/cancers17172802

**Published:** 2025-08-27

**Authors:** Amanda Reyes, Sean Maroongroge, Michelle Afkhami, Victoria Villaflor, Arya Amini, Sagus Sampath, Ellie Maghami, Thomas Gernon, Krupal Patel, Xiaochen Li, Aditya Shreenivas

**Affiliations:** 1 Department of Medical Oncology and Therapeutics Research, City of Hope National Medical Center, Duarte, CA 91010, USA; vvillaflor@coh.org (V.V.); xiaoli@coh.org (X.L.); ashreenivas@coh.org (A.S.); 2 Department of Radiation Oncology, City of Hope National Medical Center, Duarte, CA 91010, USA; smaroongroge@coh.org (S.M.); aamini@coh.org (A.A.); ssampath@coh.org (S.S.); 3 Department of Pathology, City of Hope National Medical Center, Duarte, CA 91010, USA; mafkhami@coh.org; 4 Department of Surgery, City of Hope National Medical Center, Duarte, CA 91010, USA; emaghami@coh.org (E.M.); tgernon@coh.org (T.G.); kruppatel@coh.org (K.P.)

**Keywords:** HPV+ oropharyngeal squamous carcinoma, circulating tumor DNA, minimal residual disease, TTMV-HPV DNA, head and neck carcinoma

## Abstract

**Background:** The relationship between detectable circulating tumor DNA levels and clinical outcome following definitive therapy in patients with human papillomavirus (HPV)-mediated oropharyngeal squamous cell carcinoma has not been well established. **Methods:** In this retrospective analysis of patients with HPV-positive oropharyngeal squamous cell carcinoma seen from 2016 to 2024 at a single institution, 88 patients met inclusion criteria with baseline-positive tumor tissue-modified viral HPV DNA (TTMV-HPV DNA) testing and post-treatment testing performed. **Results:** Of the 88 patients included in the survival analysis, 77 had undetectable tumor tissue-modified viral human papillomavirus DNA after treatment, while 11 had positive (detectable) tumor tissue-modified viral human papillomavirus DNA. TTMV-HPV DNA positivity after treatment was associated with worse 1-year and 2-year overall survival outcomes, at 63.5% (37.7–100, *p* = 0.022) and 50.8% (25.7–100, *p* = 0.017) compared to 100% and 96.4% (91.6–100, *p* = 0.017) in patients with undetectable TTMV-HPV DNA. Inability to clear TTMV-HPV DNA after treatment was associated with worse progression-free survival, at 45.0% (95% CI 21.8–92.7, *p* = 0.009) at 1 year and 11.3% (95% CI 1.8–71.2, *p* = <0.001) at 2 years compared to 93% (95% CI 87.3–99.1) and 84.7% (95% CI, 76.3–94.0) in patients with cleared TTMV-HPV DNA after treatment. **Conclusion:** Tumor tissue-modified viral human papillomavirus DNA positivity after definitive treatment was associated with worse survival and disease recurrence outcomes compared to that in patients with undetectable post-treatment TTMV-HPV DNA. Prospective studies are warranted to further establish the clinical utility of TTMV-HPV DNA testing and its use in surveillance, treatment intensification, or de-intensification.

## Simple Summary

In a single institution retrospective study of 88 patients, tumor tissue modified viral human papillomavirus DNA positivity after definitive treatment was associated with worse survival and disease recurrence outcomes compared to patients with undetectable (negative) post-treatment tumor tissue modified viral human papillomavirus DNA. Tumor tissue modified viral human papillomavirus DNA testing provides clinically meaningful prognostic information during surveillance after definitive treatment of HPV+ oropharyngeal cancer.

## 1. Introduction

Head and neck cancers are a heterogenous group of malignancies, though as a group, they represent a substantial proportion of cancer burden [1]. It is estimated that there are over 700,000 new cases worldwide [2]. It is estimated that 75–85% of squamous cell carcinoma head and neck (HNSCC) cancers are caused by tobacco or alcohol use, but there are increasing rates of human papillomavirus (HPV)-mediated malignancy [3]. Previous studies found that HPV-mediated HNSCC, notably oropharyngeal SCC, has a better prognosis than its non-virally mediated counterparts [4]. Oropharyngeal squamous carcinoma (OPSCC) is recognized as a distinctive entity in HNSCC [1].

Given the clinical relevance of HPV in OPSCC, HPV testing is recommended in the initial work-up for patient prognostication. However, initial pathologic testing with P16 immunohistochemistry produces false negative results [5,6]. Therefore, additional HPV testing with either deoxyribonucleic acid (DNA)/ribonucleic acid (RNA)-based testing or in situ hybridization (ISH) is indicated [5,7]. Treatment recommendations vary by disease stage and pathology type; for localized T1-2, N0-1 disease in HNSCC, patients can either receive definitive radiation or surgical resection followed by adjuvant radiation or chemo-radiation depending on the presence or absence of high-risk features [3]. For more locally advanced HNSCC (cT3-4N0M0, cT1-4N1-3M0), the standard of care is concurrent chemotherapy and radiation vs. surgery followed by adjuvant radiation or chemo-radiation depending on the presence or absence of high-risk features [3]. However, non-surgical approaches are preferred in most advanced cases unless radiation is contraindicated [8]. The optimal dosing of radiation has been investigated extensively [9,10,11], but cisplatin, if not contraindicated, has been the standard-of-care chemotherapeutic agent in combination with radiation [12]. Due to toxicity and intolerance, several dosing schedules of cisplatin have been investigated, including weekly cisplatin 40 mg/m^2^, which was non-inferior to cisplatin 100 mg/m^2^ every 3 weeks [13]. However, other regimens, including weekly cisplatin 30 mg/m^2^ and cetuximab, were inferior in even better prognosis cases of HPV + OPSCC [14,15]. Despite the intensity of these curative intent regimens, reoccurrence, whether local or metastatic, is a concern, with rates as high as 25% in HPV-positive OPSCC [4,16,17,18].

Early detection of disease recurrence is critical as early treatment intervention could improve the poor survival outcomes for patients with recurrent disease not amenable to local treatment [19]. TTMV-HPV DNA is potentially a powerful tool to achieve that aim. One retrospective study found the negative predictive value for tumor tissue-modified viral [TTMV] HPV DNA after definitive treatment in OPSCC was as high as 99.4% [95% CI 98.9–99.8] [20]. Further, the positive predictive value of detecting TTMV-HPV DNA, 3 or more months after completing treatment, was as high as 95% for recurrent disease [21]. Both of these studies employed the same assay to measure TTMV-HPV DNA levels (NavDx from Naveris Inc., Waltham, MA, USA) [4,20,21]. We performed a retrospective analysis to assess the prognostic utility of TTMV-HPV DNA clearance on patients at our institution.

## 2. Methods

### 2.1. Patient Selection and Oversight

For this retrospective analysis, electronic medical records were utilized to create a database of patients seen from 2016 to 2024 with oropharyngeal squamous cell carcinoma with appropriate IRB approval. Treatment history, primary disease location, demographics, stage at diagnosis, and programmed death-ligand 1 tumor proportion score (PD-L1 TPS) based on immunostains were also recorded. A total of 170 patients with biopsy-proven oropharynx SCC who had at least one TTMV-HPV DNA test result were identified. However, only 88 patients of the 170 patients met the inclusion criteria. In order to be included in the analysis, patients had to have baseline TTMV-HPV DNA testing prior to starting any treatment and an additional TTMV-HPV DNA test after definitive therapy. We allowed post-treatment testing within a year of completion of treatment given the limitation with retrospective analysis and delays in follow-up for various reasons. However, the vast majority of patients had testing within 3 months of treatment completion. We excluded patients who had TTMV-HPV DNA samples drawn after starting treatment even if the testing was positive to maintain consistency and accuracy across the analysis. We also excluded patients who had negative TTMV-HPV DNA prior to starting treatment, as clearance of TTMV-HPV DNA could not be analyzed in these patients. There were several patients who had baseline pre-treatment TTMV-HPV DNA testing but did not have follow-up testing for a variety of reasons including a lack of follow-up.

### 2.2. Molecular Testing of TTMV-HPV DNA

The assay from NavDx (Naveris Inc.) was used to obtain TTMV-HPV DNA levels from peripheral blood samples. The NavDx assay can distinguish between fragmented viral DNA and intact circulating viral DNA. However, the NavDx assay does not determine the HPV integration status within the cancer cells. This testing was also used to identify HPV strain, which includes the five high-risk HPV subtypes (16, 18, 31, 33, and 35). A negative TTMV-HPV DNA test, indicating no HPV DNA, detected above the threshold level of the assay was required for a patient to have cleared TTMV-HPV DNA. In this assay, patients are given a TTMV-HPV DNA score (positive, indeterminate, or negative) based on TTMV-HPV DNA per milliliter of plasma. We used the same scoring system as previous studies per protocol; positive scores included values greater than 7 (for HPV subtype 16) or greater than 12 (for HPV subtypes 18, 31, 33, or 35), indeterminate scores were between 5 and 7 (HPV 16) or 5 and 12 (HPV 18, 31, 33, or 35), and any score below 5 was considered negative [22,23].

### 2.3. Statistical Analysis

For survival analysis, median follow-up was 24.3 months (range 0.5–60.2 months). Statistical analyses were performed using R v4.1.0. Overall survival (OS) was calculated from date of diagnosis to date of death or the date of last follow-up. Progression-free survival (PFS) was calculated from the date of diagnosis to the date of disease progression/recurrence, as documented in the medical record on the date of the first imaging result with evidence of progression or last follow-up. The Kaplan–Meier method was used to estimate survival probabilities. A log-rank test was used to compare survival curves between groups. Cox proportional hazards regression was used for univariate and multivariate analyses. *p*-values < 0.05 were considered statistically significant.

## 3. Results

### 3.1. Patient Population

From this single-institution study, 170 patients were identified with OPSCC. In total, 149 of 170 (87.65%) patients were male and 21 of 170 (12.35%) were female. Overall, 88 of the 170 patients had TTMV-HPV DNA levels drawn prior to treatment with initially detectable TTMV-HPV DNA levels. The remaining 82 were either TTMV-HPV DNA-negative (either had testing performed after treatment or were HPV-negative) or did not have repeat TTMV-HPV DNA testing for analysis. Minimal residual disease (MRD) positivity was defined as a detectable level of TTMV-HPV DNA following the completion of definitive treatment in patients with detectable TTMV-HPV DNA prior to treatment. MRD-negative was defined as patients with detectable TTMV-HPV DNA prior to definitive treatment followed by no detection of TTMV-DNA levels after definitive therapy. In this cohort, the majority of patients became MRD-negative by 3 months after completion of treatment, but a smaller portion of patients took longer to become MRD-negative or follow-up testing was delayed. We reviewed these cohorts separately as well. Of the MRD-negative patients, 62 (80.5%) were HPV 16-positive compared to 10 out of 11 (90.9%) of the MRD-positive patients and 29 out of 82 (35.4%) of those with unknown MRD status (Table 1). All 8 of the HPV 33 patients, 3 of 4 HPV 35 patients, and 4 of 5 HPV 18 patients were MRD-negative (Table 1). The average age of the patients at sample collection was 64.5 (28–88), 65.0 (38–87) in the MRD-negative cohort, and 64 (30–74) in MRD-positive cohort (Table 1).

### 3.2. MRD Status

**Figure 1:**
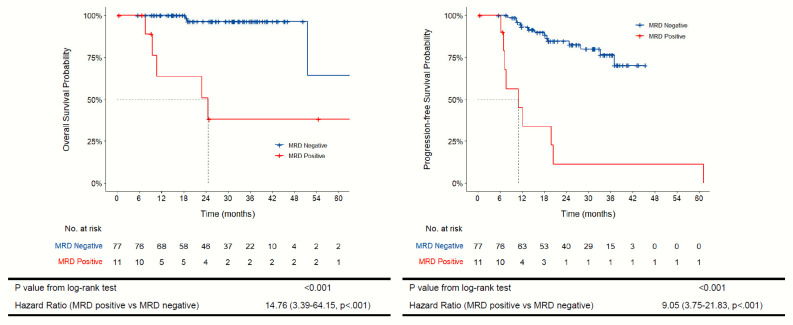
Kaplan–Meier curve for overall survival and progression-free survival by MRD status.

In the analyzed patient population, 88 patients fit the inclusion criteria above. In univariate analysis, TTMV-HPV DNA positivity, now defined as MRD-positive patients, was associated with worse 1-year and 2-year overall survival outcomes: 63.5% (37.7–100, *p* = 0.022) and 50.8% (25.7–100, *p* = 0.017) compared to 100% and 96.4% (91.6–100, *p* = 0.017) in MRD-negative patients (Figure 1 and Table 2). MRD positivity was also negatively associated with progression-free survival (PFS), with PFS rates of 45.0% (95% CI 21.8–92.7, *p* = 0.009) at 1 year and 11.3% (95% CI 1.8–71.2, *p* = <0.001) at 2 years compared to 93% (95% CI 87.3–99.1) and 84.7% (95% CI, 76.3–94.0), respectively (Figure 1 and Table 2). In multivariate analysis, MRD positivity was associated with worse clinical outcomes, with HR 4.19 (95% CI 0.57–31.00, *p* = 0.161) for OS and HR 4.43 (95% CI 1.30–15.09, *p* = 0.017) for PFS (Table 3). No other clinical factors (including advanced age, PD-L1 status, or receipt of chemoRT) were significantly associated with OS on multivariate analysis (Table 3). As there were very few patients with non-HPV subtype 16, there was limited comparison of survival outcomes between the HPV subtypes. Of note, a higher percentage of MRD-positive patients had advanced disease (stage III/IV) at diagnosis compared to the MRD-negative patients, which may have had some impact on unadjusted clinical outcomes. Further, all of the MRD-positive patients received radiation as a part of treatment, but approximately 82% of patients that were MRD-negative received radiation. Like advanced stage, this could have impacted the overall survival analysis but was not significant in multivariable analysis.

### 3.3. Rate of MRD Clearance

**Figure 2:**
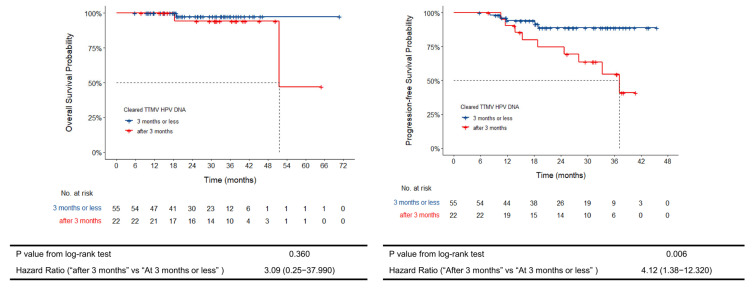
Kaplan–Meier curves for overall survival and progression-free survival by rate of MRD clearance.

We also documented the rate of MRD clearance from the start of treatment to the date of a negative TTMV-HPV DNA test result. The median time to HPV clearance was 3 months since the completion of treatment, with the earliest documented clearance at 1 month after the completion of treatment. In total, 55 patients cleared TTMV-HPV DNA at 3 months or sooner after treatment initiation, which we define as rapid MRD clearance, versus 22 patients who cleared TTMV-HPV DNA after the 3-month cut off. PFS at 1 year for rapid MRD clearance was 94.1% (95% CI 87.7–100%, *p* = 0.563) vs. 90.5% (95% CI 78.8–100%). Two-year PFS was 88.9% (95% CI 79.8–96.7, *p* = 0.196) in rapid MRD clearance vs. 74.8% (95% CI 57.8–96.7) (Figure 2). Two-year OS in the rapid clearance group was 97.3% (95% CI 92.2–100, *p* = 0.481) vs. 94.1% (95% CI 83.6–100) (Figure 2).

**Figure 3:**
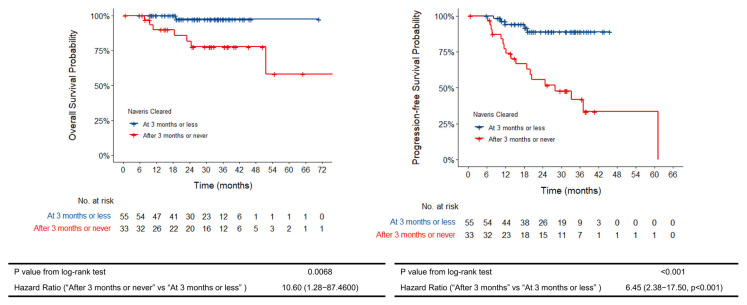
Kaplan–Meier curves for overall survival and progression-free survival by rate of MRD status at 3 months.

In a sensitivity analysis, we compared patients who were MRD-negative at 3 months (n = 55 patients) compared to those who were MRD-positive or did not have complete testing at 3 months, irrespective of whether they eventually cleared TTMC-HPV-DNA. We choose 3 months as the cutoff time given the standard-of-care practice of obtaining imaging, namely fluorodeoxyglucose-positron-emission tomography [PET] imaging, at 3 months, the optimal timing from previous meta-reviews to ensure accuracy as well as early detection of recurrence [24,25,26]. Two-year PFS for the MRD-negative patients at 3 months was significantly improved, at 88.9% (95% CI 79.8–98.7%, *p* = 0.0016) vs. 55.5% in the MRD-positive group at 3 months (95% CI 39.7–77.1%) (Figure 3). Two-year OS was also improved in the MRD-negative patients at 3 months, at 97.3% (95% CI 92.2–100%, *p* = 0.043) vs. 81.7% (95% CI 68.3–97.7%) in the MRD-positive patients (Figure 3).

## 4. Discussion

While cure rates in HPV-associated OPSCC after definitive treatment are high, clinical outcomes can be poor in patients with recurrent or metastatic disease. Therefore, early detection of disease, even before findings on imaging, is essential and could potentially aid in patient risk stratification. In our single-institution retrospective study, patients who remained TTMV-HPV DNA-positive above the threshold of detectability (MRD-positive) after definitive treatment had worse survival outcomes compared to patient’s whose TTMV-HPV DNA cleared after first-line definitive therapy. Further, looking at MRD status at 3 months after completion of definitive treatment, MRD-negative status at this time point, versus MRD-positive status at this time point, was associated with improved survival outcomes. This suggests that MRD status as soon as 3 months after definitive therapy can be considered a prognostic marker for use in clinical practice. The rate of MRD clearance at 3 months versus longer than 3 months in patients who eventually cleared the TTMV-HPV DNA did not show statistically significant results but could be due to several factors including smaller sample size. It should be noted that in our current clinical practice, TTMV-HPV DNA testing is most often repeated 3 months after completion of definitive treatment. The longest time to clearance was difficult to accurately assess given the varying times of follow-up in this retrospective analysis, as a few patients did not have repeat testing for over a year. Additionally, the timing of repeat TTMV-HPV DNA testing was not uniform, and some patients did not have repeat testing for several months after treatment. After controlling other factors, this TTMV-HPV DNA response was the only tested factor significantly associated with survival or progression-free survival outcomes.

Our study differed from previous studies, in which most of the patients did not have pre-treatment TTMV-HPV DNA levels [20]. All the patients in our MRD analysis cohort had a pre-treatment TTMV-HPV DNA level available for comparison. Recent studies revealed contrasting results when comparing survival outcomes in HNSCC by HPV subtype, namely HPV-16 versus others, as HPV-16 has been associated with worse survival and improved survival compared to the other less common subtypes [27,28]. Due to the limited number of patients with HPV subtypes in our study, there was limited statistical power comparing the two groups. However, with a larger study across multiple institutions, a difference in clinical outcomes could be appreciated by HPV subtype. It should be noted that there is a minimum threshold of detectability for TTMV-HPV and that some patients categorized as having presented with negative circulating TTMV-HPV DNA in our analysis may have had very low levels of HPV, below the level of detection, and were not truly HPV-negative [7,20,21,29]. Further, previous studies have demonstrated an association between more advanced disease, higher nodal stage, and larger tumor size and a higher detection rate [29]. These clinical characteristics were not assessed among our cohort.

While not evaluated specifically in our study, viral-negative HNSCC patients could also benefit from either early detection of reoccurrence or risk stratification after definitive treatment. To this point, a recent study found that patients with positive circulating tumor DNA (ctDNA) after definitive treatment for viral-negative HNSCC had worse PFS [HR 7.33 (95% CI 3.12–17.2; *p* < 0.001)] compared to ctDNA-negative patients [30]. With advances in laboratory techniques, the use of sensitive and specific assays for the detection of tumor cells has greatly increased. However, the optimal strategy for implementing MRD or ctDNA testing has not been fully explored and accepted into the management of OPSCC.

From our study, it is clear that MRD-positive patients, after treatment for both HPV-negative and -positive HNSCC, have a worse prognosis. These patients could benefit from earlier interventions including enrollment into a clinical trial, initiation of adjuvant systemic therapy, or closer surveillance imaging. MRD kinetics or the rate of TTMV-HPV DNA clearance after the initiation of treatment did not have a statistically significant impact on survival outcomes in our analysis, but this may be due to limitations in the retrospective design, small sample size, or shorter follow-up interval.

In clinical practice, MRD monitoring can also be utilized to identify early recurrence of disease, or potentially risk-stratify for disease recurrence when imaging is inconclusive. In our cohort of patients, this was demonstrated in a patient case of an HPV+ OPSCC patient treated with definitive chemoradiotherapy with potential recurrent disease in lymph nodes but non-specific findings on imaging. TTMV-HPV DNA testing remained negative at the time of repeat imaging. Therefore, the decision was made to repeat imaging in 1 month. On repeat imaging, the lymph nodes had resolved and were determined to be inflammatory/infectious in etiology. The patient resumed routine monitoring and continues to have no evidence of disease several months later. Because only a limited number of patients who were MRD-negative recurred, the detection of early recurrent disease could not be adequately assessed in this study.

## 5. Conclusions

Tumor tissue-modified viral human papillomavirus DNA testing provides clinically meaningful prognostic information during surveillance after definitive treatment of HPV+ oropharyngeal cancer. Prospective studies are warranted to further establish the clinical utility of TTMV-HPV DNA testing and its use in surveillance, treatment intensification, or de-intensification. Further, additional studies in the first-line metastatic or first-recurrence setting may clarify the utility of ctDNA testing in the metastatic/recurrent population.

## Figures and Tables

**Table 1:** Patient demographics.

	Overall (N = 170)	MRD-Negative (N = 77)	MRD-Positive (N = 11)	Unknown (N = 82)
Age_at_collection				
Median [Min, Max]	64.5 [28.0, 88.0]	65.0 [38.0, 87.0]	64.0 [30.0, 74.0]	64.0 [28.0, 88.0]
HPV_Subtype				
HPV 16	101 (59.4%)	62 (80.5%)	10 (90.9%)	29 (35.4%)
HPV 18	5 (2.9%)	4 (5.2%)	1 (9.1%)	0 (0%)
HPV 33	8 (4.7%)	8 (10.4%)	0 (0%)	0 (0%)
HPV 35	4 (2.4%)	3 (3.9%)	0 (0%)	1 (1.2%)
Negative	32 (18.8%)	0 (0%)	0 (0%)	32 (39.0%)
Missing	20 (11.8%)	0 (0%)	0 (0%)	20 (24.4%)
PDL1_Expression				
Mean (SD)	0.263 (0.296)	0.302 (0.316)	0.196 (0.161)	0.230 (0.286)
Median [Min, Max]	0.150 [0, 1.00]	0.200 [0, 1.00]	0.200 [0.0100, 0.400]	0.100 [0, 1.00]
Missing	70 (41.2%)	27 (35.1%)	4 (36.4%)	39 (47.6%)
Primary Site: Tonsil				
No	77 (45.3%)	32 (41.6%)	5 (45.5%)	40 (48.8%)
Yes	93 (54.7%)	45 (58.4%)	6 (54.5%)	42 (51.2%)
Primary Site: Base of Tongue				
No	114 (67.1%)	51 (66.2%)	9 (81.8%)	54 (65.9%)
Yes	56 (32.9%)	26 (33.8%)	2 (18.2%)	28 (34.1%)
Other Primary Sites				
No	74 (43.5%)	31 (40.3%)	4 (36.4%)	39 (47.6%)
Yes	96 (56.5%)	46 (59.7%)	7 (63.6%)	43 (52.4%)
Surgery				
No	134 (78.8%)	63 (81.8%)	10 (90.9%)	61 (74.4%)
Yes	30 (17.6%)	10 (13.0%)	1 (9.1%)	19 (23.2%)
Missing	6 (3.5%)	4 (5.2%)	0 (0%)	2 (2.4%)
Chemo				
No	13 (7.6%)	7 (9.1%)	0 (0%)	6 (7.3%)
Yes	150 (88.2%)	66 (85.7%)	11 (100%)	73 (89.0%)
Missing	7 (4.1%)	4 (5.2%)	0 (0%)	3 (3.7%)
Radiation				
No	15 (8.8%)	9 (11.7%)	0 (0%)	6 (7.3%)
Yes	146 (85.9%)	63 (81.8%)	11 (100%)	72 (87.8%)
Missing	9 (5.3%)	5 (6.5%)	0 (0%)	4 (4.9%)
Stage				
I	60 (35.3%)	31 (40.3%)	3 (27.3%)	26 (31.7%)
II	43 (25.3%)	25 (32.5%)	2 (18.2%)	16 (19.5%)
III	47 (27.6%)	19 (24.7%)	3 (27.3%)	25 (30.5%)
IV	17 (10.0%)	2 (2.6%)	3 (27.3%)	12 (14.6%)
Missing	3 (1.8%)	0 (0%)	0 (0%)	3 (3.7%)

**Table 2:** Univariate analysis.

Variable	N	HR for OS (95% CI)	*p* Value	HR for PFS (95% CI)	*p* Value
MRD Status					
Negative (Reference)	77	1	-	1	-
Positive	11	14.76 (3.39–64.15)	<0.001	9.05 (3.75–21.83)	<0.001
Age					
<65 years (Reference)	41	1	-	1	-
≥65 years	47	0.46 (0.11–1.91)	0.283	0.40 (0.17–0.97)	0.042
PDL1 Expression					
CPS < 20 (Reference)	27	1	-	1	-
CPS ≥ 20	30	1.00 (0.16–6.24)	0.999	0.84 (0.34–2.09)	0.705
HPV Status					
HPV16 (Reference)	72	1	-	1	-
Other	16	0.00 (0.00-Inf)	0.999	0.79 (0.23–2.68)	0.702
Treatment Type					
CRT (Reference)	69	1	-	1	-
Other	14	0.00 (0.00-Inf)	0.999	0.79 (0.23–2.73)	0.714
Stage					
I	34	1	-	1	-
II	27	0.38 (0.04–3.46)	0.393	1.21 (0.41–3.60)	0.732
III	22	0.88 (0.16–4.85)	0.886	1.63 (0.57–4.66)	0.362
IV	5	2.27 (0.25–21.07)	0.47	3.34 (0.68–16.38)	0.137

Inf: infinite.

**Table 3:** Multivariate analysis.

Variable	HR for OS (95% CI)	*p* Value	HR for PFS (95% CI)	*p* Value
MRD-Positive vs. -Negative	2.01 (0.20–20.54)	0.557	3.66 (0.91–14.73)	0.068
Age ≥ 65 vs. <65 years	0.83 (0.11–6.44)	0.861	0.40 (0.14–1.17)	0.094
PDL1 CPS ≥ 20 vs. <20	1.06 (0.14–8.06)	0.953	0.94 (0.31–2.79)	0.907
HPV Other vs. HPV16	-	-	0.85 (0.18–4.07)	0.834
Other Treatment vs. CRT	-	-	1.23 (0.31–4.78)	0.768
Stage II vs. I	0.00 (0.00-Inf)	0.999	1.09 (0.28–4.28)	0.907
Stage III vs. I	1.46 (0.18–12.07)	0.724	1.80 (0.48–6.75)	0.384
Stage IV vs. I	1.75 (0.09–32.60)	0.706	1.41 (0.21–9.47)	0.724

HR: Hazard Ratio; CI: Confidence Interval; OS: Overall Survival; PFS: Progression-Free Survival; CRT: Chemoradiotherapy; CPS: Combined Positive Score; Inf: infinite.

## Data Availability

The original contributions presented in this study are included in the article. Further inquiries can be directed to the corresponding author.
